# Metal and Polymeric Strain Gauges for Si-Based, Monolithically Fabricated Organs-on-Chips

**DOI:** 10.3390/mi10080536

**Published:** 2019-08-15

**Authors:** William F. Quirós-Solano, Nikolas Gaio, Cinzia Silvestri, Gregory Pandraud, Ronald Dekker, Pasqualina M. Sarro

**Affiliations:** 1 Department of Microelectronics, Electronic Components, Technology and Materials (ECTM), Delft University of Technology, Mekelweg 4, 2628 CD Delft, The Netherlands; 2 School of Electronics Engineering, Instituto Tecnológico de Costa Rica, A.P. 159, 7050 Cartago, Costa Rica; 3 BIOND Solutions B.V., Mekelweg 4, 2628 CD Delft, The Netherlands; 4 Electrical Sustainable Energy, Photovoltaic Materials and Devices (PVMD), Delft University of Technology, Mekelweg 4, 2628 CD Delft, The Netherlands; 5 Philips Research, High Tech Campus, 5656 AE Eindhoven, The Netherlands

**Keywords:** organ-on-chip, MEMS, silicon, PDMS, membranes, cell, strain, stress

## Abstract

Organ-on-chip (OOC) is becoming the alternative tool to conventional in vitro screening. Heart-on-chip devices including microstructures for mechanical and electrical stimulation have been demonstrated to be advantageous to study structural organization and maturation of heart cells. This paper presents the development of metal and polymeric strain gauges for in situ monitoring of mechanical strain in the Cytostretch platform for heart-on-chip application. Specifically, the optimization of the fabrication process of metal titanium (Ti) strain gauges and the investigation on an alternative material to improve the robustness and performance of the devices are presented. The transduction behavior and functionality of the devices are successfully proven using a custom-made set-up. The devices showed resistance changes for the pressure range (0–3 kPa) used to stretch the membranes on which heart cells can be cultured. Relative resistance changes of approximately 0.008% and 1.2% for titanium and polymeric strain gauges are respectively reported for membrane deformations up to 5%. The results demonstrate that both conventional IC metals and polymeric materials can be implemented for sensing mechanical strain using robust microfabricated organ-on-chip devices.

## 1. Introduction

Organ-on-chip (OOC) aims to become the alternative tool for in vitro screening. Researchers are still facing biological and technological challenges that impede this cutting-edge technology to be adopted as a routine tool in drug development. The limited scalability of current fabrication processes and the lack of self-integrated monitoring are among the technical limitations hindering its adoption [1]. Thus, monolithically microfabricated OOC devices have been developed aiming to overcome such limitations. The so-called *Cytostretch* [2,3], has been developed as a heart on chip with integrated microelectrodes. This platform enables the access of data related to the action potential generated by iPSC-derived cardiomyocytes with also the possibility to precisely stimulate electrically the cell culture.

Other heart-on-chips including mechanical stimulation have also demonstrated their advantage to promote structural organization and maturation of the cells [4,5,6,7]. Generally, most devices are based on mechanically flexible materials that enable continuous mechanical stimulation of the different cell cultures [8,9,10,11,12]. Polydimethylsiloxane (PDMS) is typically used as main structural substrate facilitating dynamic stretching, exploiting its intrinsic advantages of biocompatibility, optical transparency and mechanical flexibility [13,14,15].

Nevertheless, the lack of robustness of most devices is caused by their reliance on bulk optical microscopy and pneumatic transduction. The type and amount of data to be acquired from the cell microenvironment and biological processes is constrained. A complete view of cell behavior, particularly in heart-on-chips, might be obtained if mechanical stress, bioelectrical activities, pH level, and potassium and oxygen concentration could be quantitatively measured and controlled in situ in a spatio-temporal manner.

Unlike other works, the Cytostretch is a modular platform where robust technologies for sensing and actuation can be integrated for such purpose. The possibility to monitor strain on the substrate was preliminary demonstrated with metal strain gauges that allow indirect quantification of the mechanical stress in the flexible membrane. However, previous work did not yet fully exploit the reproducibility, reliability and robustness of the polymer-last approach [2], where complex and electrical microstructures are fabricated prior to PDMS deposition.

This paper presents the development of metal and polymeric strain gauges for in situ monitoring of mechanical strain in the Cytostretch platform. Specifically, the optimization of the fabrication process of metal titanium (Ti) strain gauges and the investigation on an alternative material to improve the robustness and performance of the devices, are presented. Polymeric strain gauges using poly(3,4-ethylenedioxythiophene)(PEDOT:PSS) are investigated to exploit the well-known electronic conduction, high gauge factor, biocompatibility, high transparency and particularly mechanical flexibility of the material [16,17,18,19,20,21].

## 2. Strain Gauges on PDMS Membranes

Strain gauges are microstructures commonly used to quantify the strain of a holding substrate subjected to mechanical stress. By monitoring the change in the electrical resistance of these microstructures, the strain experienced by the substrate when stretched can be indirectly obtained. Integrating strain gauges in a microfabricated PDMS-based OOC platform enables the acquisition and quantification of the mechanical strain provided to a cell culture, thus allowing the gathering of more relevant information for biological studies.

### 2.1. Device Concept and Design

**Figure 1:**
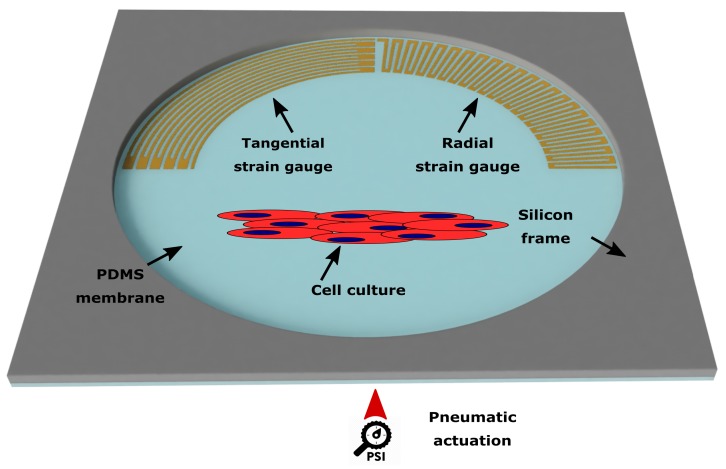
The architecture of the device investigated for stress sensing in a microfabricated PDMS-based OOC platform: Tangential and radial microstructures (strain gauges) on PDMS membranes suspended from a holding silicon frame. The membrane is pneumatically actuated to provide the stretching to a cell culture on its surface.

The devices investigated consist of either metal or polymeric strain gauges integrated on a PDMS membrane suspended from a silicon holding frame, a platform based on *Cytostretch* [2,3]. The membrane acts as a stretchable substrate for cell culturing with the strain gauges allowing a continuous electrical monitoring of the strain of the membrane when mechanically stretched. Pneumatic actuation enables the stretching of the membrane by applying a pressure load on its bottom surface. In Figure 1, the architecture of the devices is shown.

The shape, thickness and diameter of the membrane is determined based on the assumption that a semicircular profile of the radial strain is obtained in the membrane when stretched (Equation (1)), thus aiming at providing specific radial strains (0–10%) for pneumatic actuation pressures up to 2 kPa. The circular shape was preferred to exploit the symmetry of the strain distribution along the membrane [22]. The final design comprises a flexible circular 9 μm-thick (*t*) PDMS membrane of 3 mm in diameter (D). The radial strain of the membrane, $\varepsilon_{r}$, is given by:

(1) $$\varepsilon_{r}=\frac{r_{final}-2r}{2r}=\frac{{\left(r\right)}^{2}+d^{2}}{2rd}arcsin\left(\frac{2rd}{{\left(r\right)}^{2}+d^{2}}\right)-1$$

where *r* and *d* are the radius and the vertical displacement of the membrane, respectively.

The position and dimensions of the strain gauges are defined based on the expected distribution of radial and tangential strain on circular membranes. The tangential strain, $\varepsilon_{t}$, is related to the radial strain according to

(2) $$\varepsilon_{t}\left(\varrho \right)=\varepsilon_{r}\left(1-\frac{\varrho^{2}}{r^{2}}\right)$$

where ϱ is the distance from the center of the membrane. Consequently, the strain gauges were located close to the edge of the membrane, where the gradient of the tangential strain along the radial direction is high.

Serpentine-like geometries of length ($L_{r},L_{t}$) were then defined to maximize the microstructures parallel to the expected main strain directions (radial and tangential). Likewise, the width (*W* = 20 μm) was then defined to exploit the expected mechanical behavior and to have final electrical resistances in the range of kΩ, matching with standard resistors necessary for further signal conditioning thickness was defined to comply with the above mentioned electrical criteria and reduced as much as possible to minimize the effect of the strain gauges on the membrane deformation.

### 2.2. Modelling and Simulation

An initial assessment, trough numerical simulations, was performed to analyze the expected mechanical performance of the envisioned microstructures. The microstructures and the PDMS membrane showed in Figure 1 were modelled and the corresponding solution to the set of equations was obtained using the FEM-based software Comsol Multiphysics${}^{®}$. The mechanical modelling implemented was defined so that the equations can be computationally solved and particularly the boundary conditions match the experimental conditions for the intended operation of the devices and the subsequent electromechanical characterization. As demonstrated in other works, numerical simulation provides better insight on the mechanical behavior of membranes with strain gauges compared to analytical solutions. On thin membranes, the effect of the microstructures on the final deformation of the membrane is better contemplated [22] by numerical solutions.

The modelling of the membrane with strain gauges was based on both linear and non-linear equations of solid mechanics. The non-linearity is relevant for the model as it takes into account the non-elastic behavior of the polymeric membrane [23]. Therefore, it is included in the model by introducing a stress-strain curve in the material properties, based on data previously reported [23,24]. The strain gauges were modelled assuming a linear isotropic and elastic material. Values reported in the literature were used for the Youngs Modulus (90 GPa, 2 GPa) and Poisson ratio (0.31, 0.35) of titanium (Ti) and poly(3,4-ethylenedioxythiophene) polystyrene sulfonate (PEDOT:PSS), respectively [16,17,18,25].

The boundary conditions established for the model were determined based on the envisioned use of the device for mechanical stretching of cell cultures. A boundary condition of zero displacement on the substrate surface surrounding a circular membrane was considered, as this is the region where the membrane is clamped to the silicon substrate. A boundary force equivalent to the pneumatic pressure applied at the bottom surface of the membrane was included.

Subsequently, the geometry was meshed with (14 thousand elements) a high quality (≈0.83) meshing, to calculate the displacement and strain fields.

**Figure 2:**
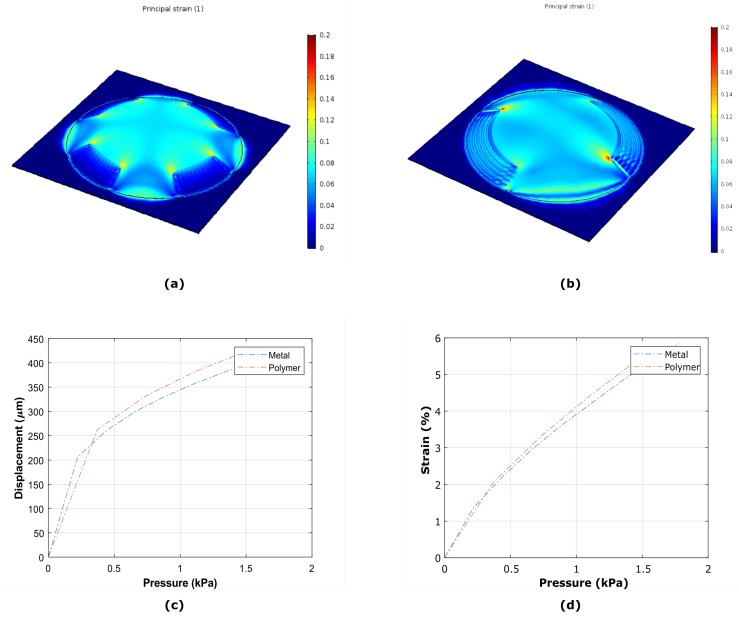
Strain field of the membranes with radial (**a**,**b**) tangential metal strain gauges for a boundary force corresponding to 2 kPa. (**c**) The curve of the displacement at the center of a membrane with metal (blue line) and polymeric (red line) strain gauges for pressures up to 2 kPa. (**d**) Corresponding average strain of the membrane.

The computation was carried out determining the displacement field of each of the elements of the geometry representing the device. In Figure 2a,b, the strain field of a membrane with radial and tangential metal strain gauges actuated with 2 kPa, is shown. In Figure 2c,d, the expected displacement at the center of the membrane and the average strain for both metal and polymeric strain gauges are reported.

## 3. Materials and Methods

### 3.1. Materials

Two materials were investigated, namely a metal, titanium, and a conductive polymer, poly(3,4-ethylenedioxythiophene)(PEDOT:PSS). Titanium was selected given its well-known mechanical behavior, biocompatibility, gauge factor (GF ≃0.8) and the possibility to pattern it with conventional lithography and dry etching techniques [26,27,28,29,30].

PEDOT is a polymer derived from ethylene dioxythiophene monomer. The electrical conductivity is caused by the delocalized π-electrons within its chemical structure and the presence of sulfonated polystyrene (PSS). It was adopted due to its known electronic conduction, biocompatibility, high transparency (≥90%) and particularly for its mechanical flexibility (E ≃ 1.2 GPa) [16,17,18], making it suitable for either sensing or stimulating microstructures [31]. Moreover, previous studies suggest that this polymer can provide a gauge factor in the range of 0.48–17.8 [19,20,21].

### 3.2. Fabrication

To fabricate the strain gauges on PDMS membranes, wafer-level fabrication processes were developed based on conventional photolithography and MEMS microfabrication techniques. The nature of the two conductive materials adopted is significantly different, resulting in the development and testing of two different processes to integrate the strain gauges.

#### 3.2.1. Metal Strain Gauges

**Figure 3:**
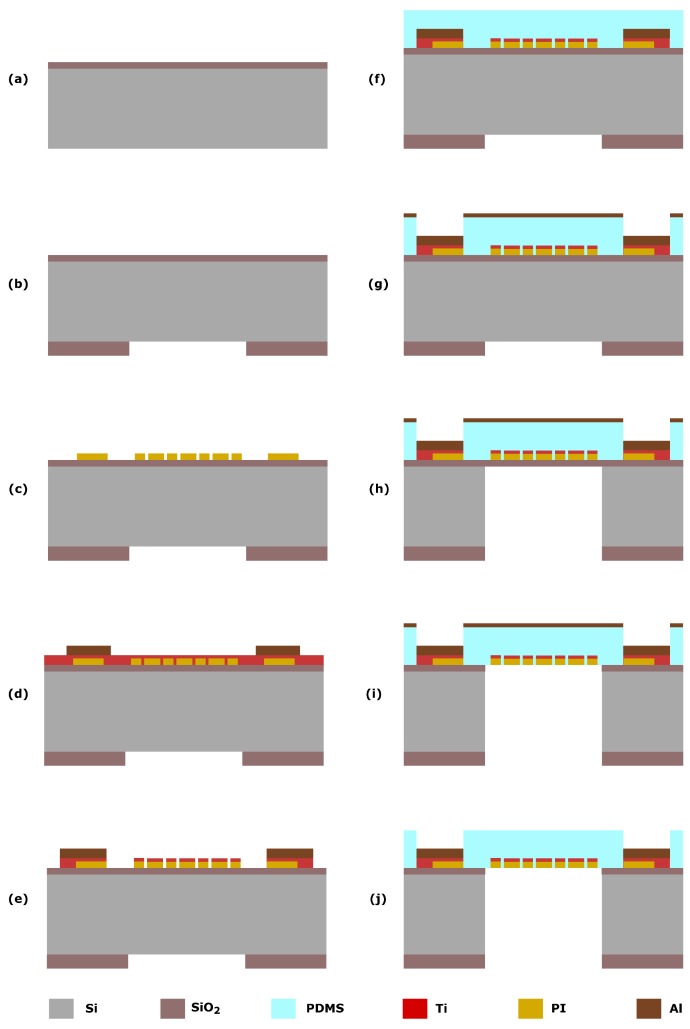
Main steps of the fabrication process for the integration of Ti strain gauges on PDMS membranes. (**a**) Deposition of oxide on the wafer front side. (**b**) Deposition and patterning of the oxide on the wafer back side to define the circular membrane. (**c**) Deposition and patterning of PI layer for electrical and mechanical isolation. (**d**) Deposition of Ti and patterning of electrical contacts (Al). (**e**) Patterning of the metal layer corresponding to the strain gauges (Ti). (**f**) Deposition of PDMS layer. (**g**) Deposition and patterning of the Al masking layer and etching of the PDMS layer to open the electrical contacts. (**h**) Etching of the silicon substrate using a DRIE process. (**i**) Removal of the landing SiO${}_{2}$. (**j**) Removal of the masking layer (Al) by wet and dry etching.

The fabrication process of the metal strain gauges is schematically depicted in Figure 3. The process starts with the deposition of a 1 μm plasma-enhanced chemical vapor deposition (PECVD) silicon oxide (SiO${}_{2}$) on the front side of a 100 mm in diameter, 525 μm-thick silicon wafer (Figure 3a). The oxide acts as an etch-stop layer for the Deep Reactive-Ion Etching (DRIE) step used to form the membrane. On the backside of the wafer, a 6 μm PECVD SiO${}_{2}$ hard-mask layer is deposited and patterned to define the circular shape membranes (Figure 3b).

Then a 300 nm photosensitive polyimide (PI) layer is deposited and patterned. This layer is included to provide electrical isolation as well as protection of the metal lines during the subsequent steps of the process (Figure 3c). Next, a 600 nm-thick Aluminum (Al) and 100 nm-thick titanium (Ti) layer are sputtered on the PI at room temperature. The resistivity of the sputtered materials is 20 μΩ-cm and 106 μΩ-cm, respectively. The Al layer is patterned and selectively etched by using wet etching (Figure 3d). After defining the contacts, the Ti layer is patterned by dry etching (ICP Plasma etcher, Trikon Omega 201) with a 2μm-thick positive resist as a masking layer (Figure 3e).

Then, a layer of PDMS (Dow Corning, Sylgard 184) is spin-coated on the front side. The elastomer and curing agent are mixed in a 10:1 ratio and degassed in a centrifugal vacuum mixing and degassing tool. The polymer is spun in three steps: a first step to spread the material over the silicon wafer at 10 rpm for 10 s; a second step for uniform spreading at 300 rpm for 20 s and a final step at 6000 rpm for 30 s to get the desired 9 μm thickness. The polymer curing is performed for 30 min at 90 ${}^{\circ }$C in a convection oven (Figure 3f). Subsequently, a 100 nm-thick Al layer is sputtered on the PDMS at room temperature. This temperature is set to avoid cracking of the PDMS layer during sputtering due to the high coefficient of thermal expansion of the PDMS (310 μm/m ${}^{\circ }$C). The metal is then patterned by dry etching (ICP Plasma etcher, Trikon Omega 201) to open the areas corresponding to the electrical contacts of the metallic microstructures (Figure 3g). The etching process used was optimized to avoid any issue caused by thermo-mechanical stress. No cracking of the layers is observed when exposing the materials to the thermal gradients during resist deposition and developing steps. Finally, the silicon substrate is etched from the backside by DRIE using a Bosh-based process (Figure 3h). The oxide stop layer is removed by a combination of wet and dry etching (Figure 3i). The Al layer is selectively removed by wet etching in a solution of acetic, phosphoric and hydrofluoric acid (Figure 3j).

#### 3.2.2. Polymeric Strain Gauges

Likewise, to fabricate the polymeric (PEDOT:PSS) strain gauges on PDMS membranes, a wafer-level fabrication process was developed based on conventional photolithography and MEMS microfabrication techniques. The steps for the membrane fabrication are similar to the previously described with variations specifically meant to introduce the PEDOT:PSS microstructures.

**Figure 4:**
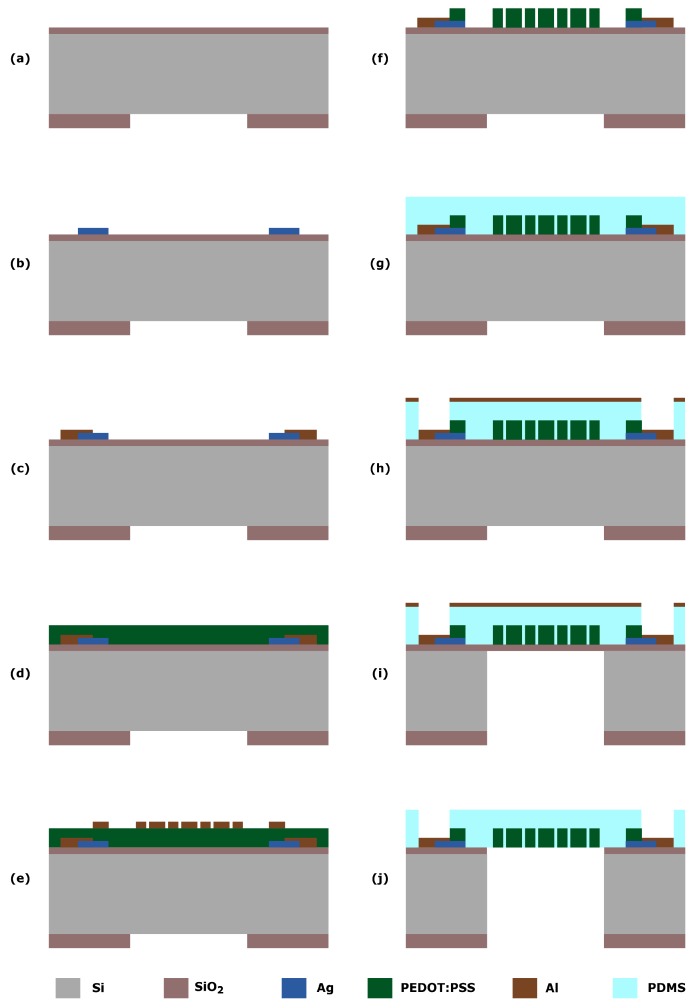
The main steps of the process flow developed for the wafer-scale fabrication of polymeric strain gauges. (**a**) Front and back deposition of the SiO${}_{2}$ oxide and patterning to define the membranes area. (**b**) Deposition of SiO${}_{2}$ and Ag on the front side patterning to define the electrical contacts. (**c**) Deposition and patterning of the Al layer to open the electrical contacts to the conductive polymer and to protect the remaining Ag layer for the subsequent etching steps. (**d**) Deposition and curing of the PEDOT:PSS layer. (**e**) Deposition and patterning of the Al masking layer. (**f**) Dry etching of the PEDOT:PSS and removal of Al masking and protective layer from the patterned strain gauges. (**g**) Deposition of the PDMS layer. (**h**) Deposition and patterning of the metallic (Al) masking layer and the PDMS layer to open the electrical contacts. (**i**) Etching of the silicon substrate using a Bosh-based DRIE process. (**j**) Removal of the landing oxide layer and the masking layer (Al) by wet and dry etching.

A 1 μm-thick PECVD SiO${}_{2}$ is deposited on a 100 mm-Si wafer. Then a layer of 100 nm of Silver (Ag) is deposited by evaporation and subsequently patterned by lift-off to create electrical contacts (Figure 4a). An Al layer is then deposited and patterned to be used as a protective layer during the subsequent polymer etching (Figure 4b). The PEDOT:PSS is deposited by spin coating and cured in an oven at 150 ${}^{\circ }$C for 40 min (Figure 4c). On top of the PEDOT:PSS another Al layer, used as a hard mask during the reactive-ion etching (RIE: O${}_{2}$, 20 mTorr, 50 W), is sputtered and patterned (Figure 4d,e). The PEDOT-based microstructures are now defined, and metal contacts are exposed. Lastly, the Al masking and protective layer are removed by wet etching using a solution of acetic acid, nitric acid and hydrofluoric acid (PES) (Figure 4f).

Once the conductive polymer is patterned (Figure 4a–f), a PDMS layer is deposited by a two-step spin coating process (Figure 4g). To open the contact pads, the PDMS is etched by RIE, and the SiO${}_{2}$ as well as the Al are etched as described for the metal strain gauges (Figure 4h–j).

### 3.3. Characterization Set-Up

**Figure 5:**
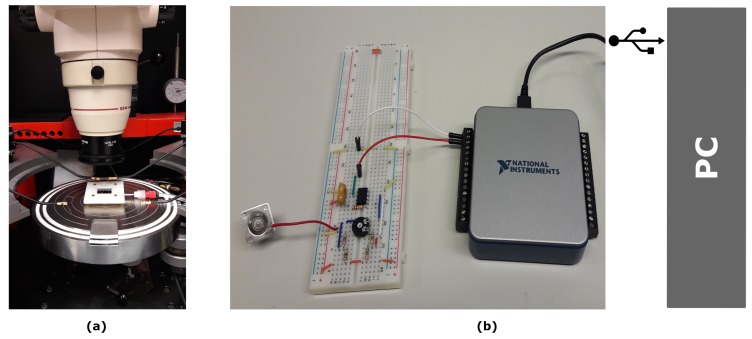
Measurement set-up developed to characterize the microfabricated strain gauges. (**a**) The electrical signal from the strain gauges is acquired by probing them with a standard probe station. The pneumatic actuation is provided simultaneously through a special coupling holder connected to a pressure source. (**b**) The electrical signal is conditioned and transmitted to a PC for further calculations and data processing.

The characterization set-up used to measure the resistance change of the strain gauges subjected to mechanical stress is shown in Figure 5. It consists of three main modules: a 3D-printed holder to interface the silicon chip with a pressure controller, a probe station and external circuitry for data acquisition and signal conditioning.

To couple both the electrical functionality and pneumatic actuation of the devices, a custom-made holder was specifically designed and fabricated by 3D printing. The holder allows for stretching the membrane through the silicon cavity by connecting the chip to a commercial pneumatic pumping system. A commercial Dual AF1 microfluidic pressure and a vacuum pump (Elveflow${}^{®}$, Biotechnology Company, Paris, France) were used to control the pressure. This system makes possible to accurately control the pressure from 0 up to 30 mbar. Moreover, the holder enables electrical connection from the chip to external circuitry as they were both designed such that the Al contact pads on the silicon substrate are easily accessed from the top.

The external circuitry to measure the resistance change is connected to the metallic and polymeric strain gauges contacts through a standard probe station. A Wheatstone bridge is implemented as a first stage for signal conditioning. For this circuit topology, an expression for the resistance of the strain gauges ($R_{SG}$) as a function of the voltage differences (*V*,$V_{CC}$) and the other resistors can be obtained by applying Kirchhoffs voltage law.

The signal (*V*) is amplified by an operational amplifier (AMP04F, Analog Devices, Norwood, MA, U.S.) with high gain (G=100) and input impedance (in the order of MΩ). The output signal of the amplifier is acquired through an Analog-to-Digital converter (USB-6001, National Instruments, Austin, TX, U.S.), enabling the direct acquisition of the data in a personal computer and the corresponding calculation of the resistance change. The signal is further processed and filtered using a digital low pass filter with the cut-off frequency fixed at 10 Hz. The cut-off frequency is set as low as possible to reduce the high-frequency noise and keep the measurement bandwidth within the expected range of typical biological processes e.g., heart rate: 1–4 Hz.

## 4. Results

### 4.1. Microfabrication

#### 4.1.1. Metal Strain Gauges

**Figure 6:**
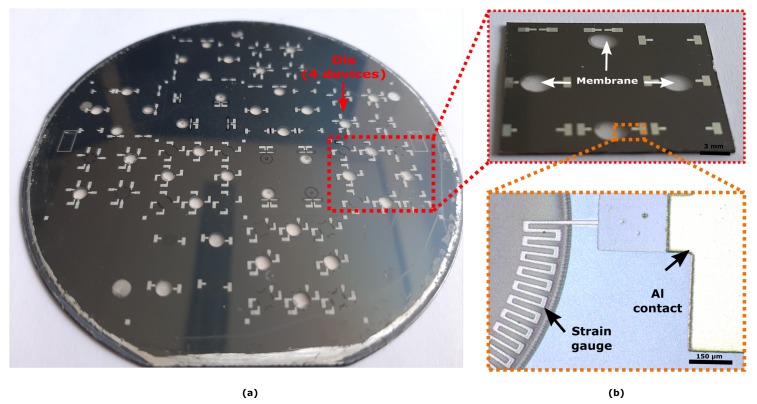
(**a**) A completed wafer containing 36 membranes equipped with strain gauges. (**b**) An optical image of a die containing four devices (**top**) and a close-up of the released membranes with Ti gauges (**bottom**). Scale bars: 150 μm (**bottom**), 3 mm (**top**).

In Figure 6 a full wafer containing several membranes with strain gauges is shown, demonstrating the wafer-scale capability of the developed process. A close-up of the released membranes (red dash line) and the titanium strain gauges can be also observed. In the background, several fibers are noticed corresponding to the supporting substrate, showing the transparency of the PDMS membrane.

#### 4.1.2. Polymeric Strain Gauges

**Figure 7:**
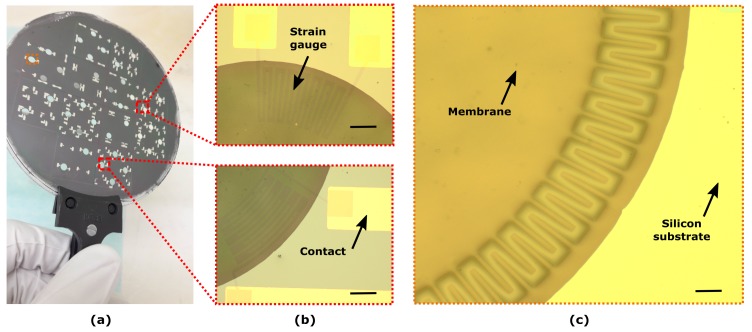
(**a**) A completed wafer containing 36 membranes equipped with polymeric strain gauges. (**b**) Optical images of radial (**top**) and tangential (**bottom**) polymeric strain gauges embedded in 10 μm-thick PDMS. (**c**) A zoom-in perspective illustrating the polymeric strain gauges integrated into the PDMS membranes. Scale bar: 100 μm.

Several membranes with polymeric strain gauges were realized on a single wafer. In Figure 7 optical images show a close-up of the polymeric strain gauges integrated on the membranes. Both radial (Figure 7a) and tangential (Figure 7b) geometries were realized, so to investigate the electromechanical response of the polymeric strain gauges.

Although PEDOT:PSS has already been applied in related devices for neuron cell study, either rigid materials were used as the supporting substrate or fabrication methods lack of compatibility with high scale manufacturing schemes [31], contrary to the advantageous processes here proposed.

### 4.2. Electromechanical Characterization

The characterization of the devices was carried out by continuously monitoring the electrical resistance at various stationary pressures. In particular, radial and tangential strain gauges, close to the end PDMS membrane edge, were characterized. The response of the devices was investigated for both Ti and PEDOT:PSS strain gauges.

#### 4.2.1. Electrical Resistance

**Figure 8:**
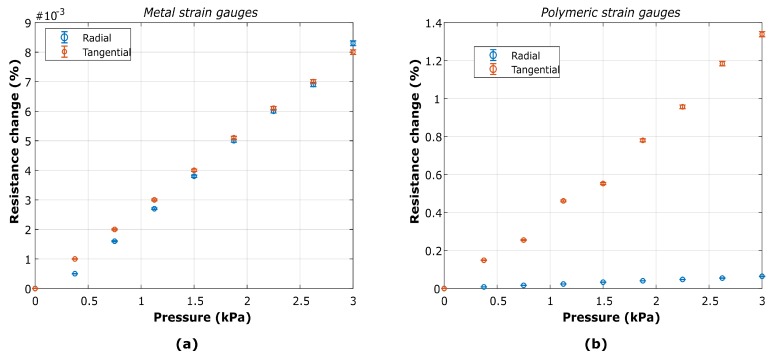
Stationary measurements of resistance change for radial and tangential strain gauges made of (**a**) titanium (Ti) and (**b**) PEDOT:PSS. Error bar: 5%.

The resistance change in stationary regime for both tangential and radial strain gauges are reported in Figure 8. The pressure increased from 0 to 3 kPa in steps of approximately 350 Pa, a value slightly higher than the minimum stable change in pressure achieved with the commercial pumping system employed.

An incremental variation of the resistance was observed. For titanium strain gauges, a relative resistance change up to ≈0.008 % over the tested pressure range was measured, for both radial and tangential geometries (Figure 8a).

For PEDOT:PSS strain gauges, a relative resistance change up to ≈1.4% was measured for the tangential geometries, a much higher value than the observed for its metal counterpart. In the case of the radial strain gauge, the resistance change was found to be ≈0.08% (Figure 8b). The measurements were performed under controlled humidity conditions with a relative humidity of ≈48%. Having a controlled humidity is important as it has been shown that both electrical and mechanical properties of PEDOT:PSS vary with humidity [16]. All results were thus obtained under the same environmental conditions. For both geometries and materials, measurements at higher pressure were not possible as the pumping system reached the maximum flow capacity at 3 kPa.

#### 4.2.2. Membrane Displacement and Strain

**Figure 9:**
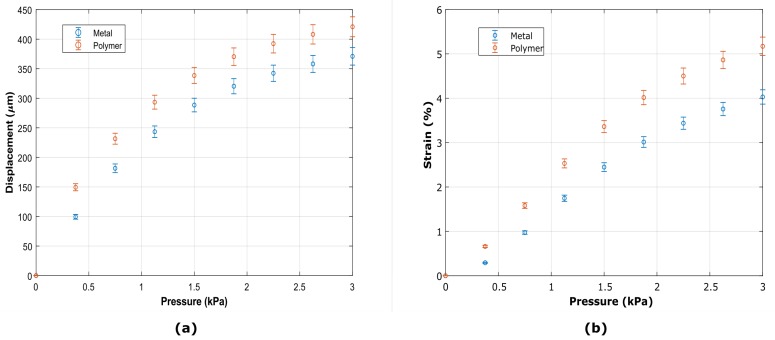
(**a**) Displacement in the center of the membranes with metal and polymeric strain gauges measured optically for different pressures set through the pumping system (1–3 kPa). (**b**) Estimation of the radial strain of the membranes with strain gauges based on the displacement measurements. Error bar: 6%.

The displacement at the center of the membrane with polymeric and metal strain gauges was measured for the same pressure ranges. The mechanical stimulus can thus be translated as strain at the surface of the material. For the microfabricated device, the strain can be approximated based on the maximum displacement of the center of the suspended polymer layer [2,3]. In Figure 9a, the displacement for different pressures is shown.

The strain on the membrane was also indirectly obtained assuming a semicircular profile and a continuous distribution along the radius. In Figure 9b, the calculated radial strain for the same pressures range used to investigate the resistance change, is shown.

#### 4.2.3. Calibration Curves

**Figure 10:**
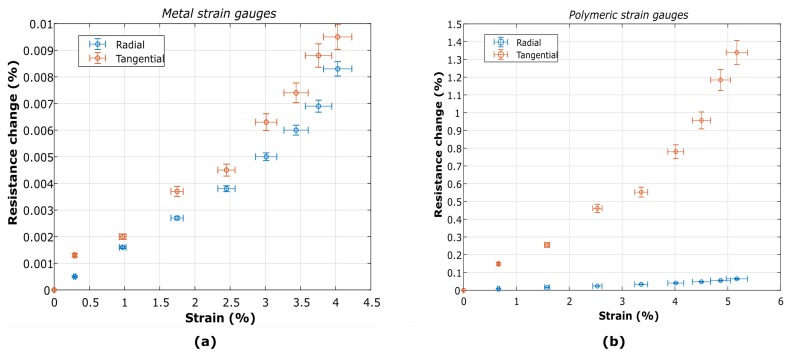
Resistance change as function of the strain on the membrane for radial and tangential strain gauges made of (**a**) titanium (Ti) and (**b**) PEDOT:PSS.

The data acquired by the electrical and mechanical characterizations (Figure 8 and Figure 9 have been used to calibrate the measured strain gauges, thus achieving the resistance change of the devices as a function of the total membrane strain. In Figure 10 the calibration curves are shown for both geometries and materials investigated.

Based on the experimental data, given a resistance change it is possible to establish an approximate transfer function for the actual strain on the membrane. The results of the Ti strain gauges suggest an estimated sensitivity of 4.5 mΩ·μm${}^{-1}$ and 11.6 mΩ·μm${}^{-1}$ for radial and tangential geometries, respectively. The tangential geometry showed higher sensitivity, confirming what suggested by theoretical and numerical analysis reported in other works [22]. For polymeric strain gauges, sensitivity of 0.571 Ω·μm${}^{-1}$ and 62 Ω·μm${}^{-1}$ for radial and tangential geometries, were obtained, respectively.

## 5. Discussion

The fabrication processes presented in this paper enabled the development of both metallic and polymeric strain gauges as a potential transduction mechanism for in situ monitoring of strain on PDMS membranes for OOC applications. The electrical resistance of the metallic strain gauges at the end of the fabrication was stable and did not show to be affected by the process. A slight deviation of approximately 2% compared to the designed values was measured. Both radial and tangential geometries (Figure 6b) did not suffer any mechanical disruption after the releasing of the membrane, a critical step to realize the final devices. This demonstrated the robustness and reliability of the fabrication process developed.

The experimental data of resistance change for both metallic radial and tangential devices are within the same order of magnitude, as can be observed in Figure 8. The results show a linear behavior up to 3 kPa, the maximum pressure measurable and supplied by the pneumatic system. However, the data on the displacement shows a non-linear tendency for the same pressure range (Figure 9), causing the non-linearities observed in the calibration curve for strains above 3% (Figure 10). This is due to the saturation of the displacement at the center of the membrane, which might be explained by the difference of a few orders of magnitude in stiffness between the membrane and the metal. Despite these non-linearities, a first linear approximation can be made to establish a transfer function for the actual strain on the membrane given a certain resistance change. Thus, the estimated sensitivity for radial and tangential geometries for strains below 3% is 4.5 mΩ·μm${}^{-1}$ and 4.4 mΩ·μm${}^{-1}$, respectively,

For polymeric strain gauges, the process was more challenging given the nature of the material. The main challenge encountered was to enable and optimize the electrical contact. As it was not possible to establish ohmic contact using readily available metals (Al, Ti, TiN), the process needed to be adapted and optimized. To do so, it was redesigned to minimize long exposure of the materials to water as PEDOT:PPS is highly hygroscopic and degraded easily in contact with water. Given its characteristic reduction potential ($E^{0}=+0.8$ V) and the accessibility to Ag deposition and patterning techniques, this material was used to create the electrical contacts. A low reactive metal is adopted due to the high acidity of the sulfonate functional group (PSS), which easily oxidizes most metals used in IC fabrication processes, unavoidably increasing the contact resistance [32]. The addition of such material increased the number of steps and complexity of the process as the material is not standard in the facilities available. Thus, Al was introduced as masking and protection layer so that the Ag contacts were never open when performing the lithography and dry etching steps, safeguarding the processing tools from possible exposure to Ag or unexpected back-sputtering.

Another addressed aspect was the temperature and baking time of the polymer. The effect of these parameters on the electrical contact was investigated. By increasing the baking temperature to 150 ${}^{\circ }$C and the baking time to 30 min, the contact resistance decreased by 20% compared to initial experiments. This indicates the importance of complete removal of water from the polymer to enhance electrical contact. It is worth mentioning that the measurements and fabrication were always carried out under controlled humidity conditions with relative humidity around 48%. Having a controlled humidity was important to keep the consistency of the experiments, as it has been shown that both electrical and mechanical properties of PEDOT:PSS might vary with humidity [22].

Regarding the electrochemical characterization of the polymeric strain gauges, the results also showed a measurable change in resistance of the microstructures when stretching the membrane. In particular, a higher value of relative resistance change (up to 1.4%) was observed compared to metal strain gauges. The tangential structures showed a higher value than the radial ones. This might be correlated with the nature of PEDOT:PSS and the deposition technique used. The polymer has a structure of fiber-like chains with de-localized π−electrons that enable the conductivity. This suggests that the deposition technique might be inducing a radial arrangement of the fibers as consequence of the spinning, leading to higher resistance changes when stretched. Significant degradation of the devices was observed with stretching and time, suggesting the need for optimizing the patterning of PEDOT:PSS microstructures and to properly characterize the dependence of mechanical properties with environmental conditions. Alternative depositions, such as electro-deposition and encapsulation of the material (e.g., Parylene, Polyimide), can be explored to address these issues and to optimize the polymer deposition, as reported in several works in implantable applications [33,34,35,36].

However, it is possible to establish a first linear approximation for the transfer function of polymeric strain gauges. In this case, the sensitivity of 0.571 Ω·μm${}^{-1}$ and 62 Ω·μm${}^{-1}$ for radial and tangential geometries, are obtained respectively for strains below 3%. This indicates a much higher sensitivity for polymeric strain gauges.

In the characterizations performed for both the polymeric and metal strain gauges, more than 50 samples per data point were collected for the mechanical and electrical measurements, with corresponding standard deviations in the 4–5% range, which suggest a high reproducibility and reliability of the devices.

## 6. Conclusions

So far, the majority of OOCs have relied entirely on bulk optical techniques (immunofluorescence end-point detection, microscope cell imaging) to acquire and analyze the information of cell microenvironments. A complete view of cell responses can be obtained if mechanical strain and other relevant cues could be quantitatively measured in situ and in a spatio-temporal manner. This paper reports wafer-scale microfabrication processes that enable the integration of metal and polymeric strain gauges in monolithically fabricated organs on chips.

The results indicate that both conventional IC metals (Ti) and polymeric materials (PEDOT:PSS) can be used for sensing mechanical strain on flexible substrates for organ-on-chip applications. The transduction behavior and the functionality of the devices were proven. A custom-made set-up allowed to show a resistance change of the devices for different pressures applied to the membranes, demonstrating the functionality of the proposed device. Relative resistance changes of approximately 0.008% and 1.2% for titanium and polymeric strain gauges have been observed, respectively for pressures up to 3 kPa applied to stretch the membranes. Correspondingly, the displacement measurements showed that for such resistance changes a strain of up to ≈5 % is induced on the membrane. The results indicate a much higher sensitivity for polymeric strain gauges and a high level of reproducibility.
